# Temperature amplifies the effect of high CO_2_ on the photosynthesis, respiration, and calcification of the coralline algae *Phymatolithon lusitanicum*

**DOI:** 10.1002/ece3.5560

**Published:** 2019-09-13

**Authors:** Laura Sordo, Rui Santos, Isabel Barrote, João Silva

**Affiliations:** ^1^ Marine Plant Ecology Research Group, Centre of Marine Sciences (CCMAR) University of Algarve Faro Portugal; ^2^ Portuguese Institute of Ocean and Atmosphere (IPMA) Olhão Portugal

**Keywords:** calcification, CO_2_, Coralline algae, heat wave, maërl, ocean acidification, photosynthesis, respiration, temperature

## Abstract

The combination of ocean acidification (OA) and global warming is expected to have a significant effect on the diversity and functioning of marine ecosystems, particularly on calcifying algae such as rhodoliths (maërl) that form extensive beds worldwide, from polar to tropical regions. In addition, the increasing frequency of extreme events, such as heat waves, threatens coastal ecosystems and may affect their capacity to fix blue carbon. The few studies where the simultaneous effects of both temperature and CO_2_ were investigated have revealed contradictory results. To assess the effect that high temperature spells can have on the maërl beds under OA, we tested the short‐time effects of temperature and CO_2_ on the net photosynthesis, respiration, and calcification of the recently described species *Phymatolithon lusitanicum*, the most common maërl species of southern Portugal. Photosynthesis, calcification, and respiration increased with temperature, and the differences among treatments were enhanced under high CO_2_. We found that in the short term, the metabolic rates of *Phymatolithon lusitanicum* will increase with CO_2_ and temperature as will the coupling between calcification and photosynthesis. However, under high CO_2_, this coupling will favor photosynthesis over calcification, which, in the long term, can have a negative effect on the blue carbon fixing capacity of the maërl beds from southern Portugal.

## Graphical abstract

In a short term, the effect of increasing CO_2_ on the metabolic rates of *Phymatolithon lusitanicum* will be accentuated with increasing temperature.

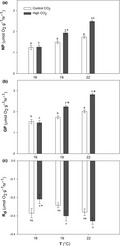

## 1 INTRODUCTION

Between 1750 and 2000, the world's oceans have absorbed about one‐third of all the carbon dioxide (CO_2_) emitted by humans (Zeebe & Ridgwell, 2011). At an unprecedented rate in geological history, atmospheric CO_2_ has risen from 280 ppm in 1750 to more than 400 ppm in 2016 (Betts et al., 2018). This absorbed CO_2_ has led to a pH drop from ~8.2 to 8.1 and to decreases in the carbonate ion concentration (CO_3_^2−^) and in the CaCO_3_ saturation state, along with an increase of bicarbonate ions (HCO_3_^−^) (Zeebe & Ridgwell, 2011). As a consequence of increasing anthropogenic emissions, the oceans have become warmer and more acidic with unknown consequences for calcifying organisms and serious implications for marine ecosystems (Johnson & Carpenter, 2012; van der Heijden & Kamenos, 2015). In addition, an increase in the frequency of extreme events, such as heat waves, threatens coastal ecosystems and their capacity to fix blue carbon (Arias‐Ortiz et al., 2018). Under global warming, heat waves are becoming more frequent and extreme. This is expected to have an irreversible impact on marine organisms and push coastal ecosystems to the limits of their resilience (Frölicher, Fischer, & Gruber, 2018). However, there is an important gap of information on the effect that these rapid temperature changes will have on the metabolism of temperate maërl beds.

Maërl beds are worldwide‐distributed aggregations of free‐living coralline algae especially sensitive to ocean acidification due to the high solubility of their high‐Mg calcite skeletons (Martin, Cohu, Vignot, Zimmerman, & Gattuso, 2013). This is the most soluble polymorph of CaCO_3_, 50% more soluble than calcite and 20% more soluble than aragonite (Ragazzola et al., 2013). Different studies on the influence of ocean acidification (OA), rise of temperature and sediment burial have confirmed a negative effect on high‐Mg calcite skeletons, suggesting that a combination of these physical stressors can be severely detrimental to coralline algae and the associated fauna and flora (reviewed in Hernandez‐Kantun et al., 2017). Previous studies have found that the calcification rates of coralline algae can decrease (Noisette, Duong, Six, Davoult, & Martin, 2013; Noisette, Egilsdottir, Davoult, & Martin, 2013), increase (Kamenos et al., 2013), or not being affected by high CO_2_ (Martin et al., 2013). As well, the photosynthetic response of coralline algae to high CO_2_ can be positive, negative, or parabolic (Martin et al., 2013; Martin & Hall‐Spencer, 2017), while respiration appears to be unaffected by high CO_2_ (Martin et al., 2013; Noisette, Duong, et al., 2013; Noisette, Egilsdottir, et al., 2013) but positively affected by temperature (Martin et al., 2013; Martin & Hall‐Spencer, 2017 for review). Because of the great variability in the results presented by different studies (Martin & Hall‐Spencer, 2017; McCoy & Kamenos, 2015), the future response and resilience of coralline algae to global change is not yet fully understood (Kroeker, Kordas, Crim, & Singh, 2010; Martin et al., 2013).

The effect of ocean acidification is exacerbated with warming (Martin & Hall‐Spencer, 2017), and some authors have found significant effects of CO_2_ on the calcification of algae only when in combination with high temperature (Martin & Gattuso, 2009). According to Vásquez‐Elizondo and Enríquez (2016), in highly illuminated ecosystems such as coral reefs, the physiology of coralline algal is more adversely affected by temperature than by high CO_2_. However, a few studies have investigated the simultaneous effect of temperature and high CO_2_ on the photosynthesis, respiration, and calcification of coralline algae (Martin et al., 2013; Noisette, Duong, et al., 2013; Vásquez‐Elizondo & Enríquez, 2016), and most available information concerns warm‐water beds while high latitude beds have received less attention in this context (McCoy & Kamenos, 2015). More information on temperate rhodolith beds is necessary to understand how these processes are related (Vásquez‐Elizondo & Enríquez, 2016), and how they depend on the interaction of temperature and CO_2_ (see Martin & Hall‐Spencer, 2017 for review).

Photosynthesis, calcification, and respiration induce changes in seawater pH and carbonate chemistry (Hurd, Hepburn, Currie, Raven, & Hunter, 2009). Both photosynthesis and respiration are suggested to control the formation of CaCO_3_ crystals on the cell walls of coralline algae. Photosynthesis increases pH while respiration decreases pH and the saturation states of calcite and aragonite, thus hindering calcification (Hurd et al., 2009; Kamenos et al., 2013; Martin et al., 2013). In the short term, calcifying photosynthetic organisms can act as a CO_2_ source through calcification and respiration, and a CO_2_ sink through photosynthesis and CaCO_3_ dissolution. On the other hand, in the long term, the accumulation of coralline algae structures over geological timescales represents a standing inorganic carbon deposit, with a carbon sink storage potential of 1.6 × 10^9^ tons per year (van der Heijden & Kamenos, 2015). Therefore, the contribution of an ecosystem to the global carbon cycle is a consequence of the balance between carbonate precipitation and dissolution, and between inorganic carbon uptake and release by photosynthesis and respiration, respectively (Gattuso, Frankignoulle, & Smith, 1999). Nonetheless, under OA, photosynthesis in calcifying algae is not likely to increase, and a greater CO_2_ and HCO_3_^‐^ availability may uncouple photosynthesis‐calcification reactions with unknown repercussions for the whole ecosystem (Koch, Bowes, Ross, & Zhang, 2013).

Global warming and OA are projected to have significant impacts in the benthic flora and coastal ecosystems of the northeast Atlantic. Brodie et al. (2014) predicted that global warming will eliminate the kelp forests from the south and ocean acidification will hamper the maërl beds from the north. As calcite saturation state drops and the sea surface isotherms move polewards, Arctic waters that are corrosive to carbonate are spreading south. Because of this, northern maërl beds are expected to be lost in the near future, while maërl beds from southern Portugal are expected to persist for longer (Brodie et al., 2014).

The information available on this geographical area is restricted to descriptive studies on the morphology and composition of maërl beds (Carro, López, Peña, Bárbara, & Barreiro, 2014; Peña et al., 2015) and its associated flora (Peña & Bárbara, 2013), plus a study where the metabolic rates of the algae were assessed at different temperatures, and the abiotic conditions of the beds were monitored for a 2‐year period (Sordo, Santos, Barrote, Freitas, & Silva, in press). Even if the studied maërl bed is under relatively stable conditions, unusual high temperature and irradiance conditions were recorded during a sporadic heat wave. Currently, there is no information on the effect that a short‐term increase of temperature could have on the metabolism of *P. lusitanicum* under high CO_2_ conditions. So far, only two studies (Sordo, Santos, Barrote, & Silva, 2018; Sordo, Santos, Reis, Shulika, & Silva, 2016) have addressed the effect of high CO_2_ on the recently identified new species *Phymatolithon lusitanicum*, the most common species found in the maërl beds from southern Portugal (Peña et al., 2015). The objective of this work is to investigate the short‐term effect of high temperature on the photosynthesis, respiration, and calcification of the free‐living coralline alga *Phymatolithon lusitanicum* under a future high CO_2_ scenario. We hypothesized that the effect of high temperature on the metabolic rates of *P. lusitanicum* will be accentuated under high CO_2_ conditions.

## 2 MATERIAL AND METHODS

### 2.1 Biological material

Rhodolith beds in southern Portugal are mainly composed by nongeniculate free‐living red coralline algae without a shell or pebble core. *Phymatolithon lusitanicum* forms the largest rhodoliths with the thickest branches in Algarve (Carro et al., 2014) and has been recently identified as a new species by Peña et al. (2015). Algae were collected by SCUBA diving in Armação de Pêra (N 37°011′0.650″/W −8°19′0.034″) the 25th of September 2016, immediately transferred to a growth chamber at field temperature (~16°C) and PAR irradiance (~20 μmol m^−2^ s^−1^) conditions for 1 day. Then, thalli were gently cleaned of epiphytes and transported to the experimental system installed in Ramalhete Marine station.

### 2.2 Experimental setup and environmental conditions

The experimental setup used in this experiment is largely based on the one described in Sordo Santos Reis Shulika and Silva (2016) and Sordo et al. (2018), where CO_2_ is controlled via direct analysis of *p*CO_2_ in seawater. Following the recommendations of Cornwall and Hurd (2015), the system was upgraded to eliminate any concerns of pseudoreplication.

Seawater is pumped from a coastal lagoon adjacent to Ramalhete Marine station and passes through a preliminary mechanical filtration (sand filters), two in‐line cartridge filters of 10–20 µm and 5 µm and two UV filters of 16 and 8W. The experimental aquaria are installed in an isolated room with controlled light and temperature conditions. For this experiment, 30 3 L experimental aquaria were connected to individual 5 L header tanks (one header tank per aquarium, *n* = 5 sets per experimental treatment). The experimental aquaria were randomly interspersed to minimize the effect of other factors. This is an open system where seawater is distributed from the header tanks into the aquaria at a flow rate of 150 ml/min. The aquaria were regularly cleaned to control the growth of turf algae.

High CO_2_ air was prepared in a large‐volume premix tank (4,000 L) where industrial grade CO_2_ was mixed with air to obtain the target CO_2_ value. This mixture was continuously prepared and injected in the header tanks. CO_2_ in the premix tank was controlled via direct analysis of CO_2_ using an infrared gas analyzer (IRGA) (WMA‐4; PPSystems) coupled to a PID controller (PID330; TEMPATRON) that operated a solenoid valve to regulate the CO_2_ flush into the premix tank.

In a previous study (Sordo et al., in press), the environmental conditions at the studied maërl bed (located in Armação de Pêra, southern Portugal, at 22 m depth) were measured in a continuous basis during more than 2 years. In this period, temperature ranged from 14 to 17°C and irradiance from 6 to 67 μmol m^−2^ s^−1^. However, a heat wave was registered in the summer of 2013, when the highest temperatures (from 17 to 23°C) and irradiance levels (62 to 73 μmol m^−2^ s^−1^) were recorded. According to NOAA (2013), in July 2013 global temperatures were higher than the average, and this was the sixth warmest July since records began in 1,880. Based on these data, the temperature levels chosen for this study were 16°C (the average temperature at the bed), 19°C (an above‐average value) and 22°C (an extreme value recorded only once during the heat wave of July of 2013).

Air temperature inside the room was controlled via an AC apparatus. Water temperature was controlled using thermostatic water chillers (Sunsun HYH‐0.5 D‐C) and additional water heaters when required. Algae were acclimated gradually to intermediate and high temperatures (19 and 22°C). Photoperiod was adjusted to 10:14 (light:dark, hr), according to the natural fluctuations recorded in autumn at the studied maërl bed. Ambient light was provided by fluorescent tubes (Osram Luminux Plus Daylight L18W/11‐860) installed above the aquaria. Irradiance was gradually increased and set to 50 μmol photons m^−2^ s^−1^ (PAR), an intermediate light level to the values recorded under natural conditions in the field. Prior to the initial measurements, thalli of similar size and morphology were selected, gently cleaned of epiphytes and distributed in a thin layer among the experimental aquaria at the experimental temperature and irradiance levels. Then, the CO_2_ was increased gradually and half of the algae were exposed to high CO_2_ conditions. In total, algae were acclimated at three different temperatures (16, 19 and 22°C) for an initial period of 15 days, followed by another 15 days where two *p*CO_2_ levels were also imposed, a control level at 400 µatm and a high CO_2_ one at 1,000 µatm.

Temperature in the aquaria was monitored using HOBO temperature loggers (Onset Corp.). Salinity (CO310 conductivity meter, VWR), pH (Orion 8103SC pH meter; Thermo scientific), and temperature (Roth digital thermometer; Hanna) were measured regularly in each aquarium. The *p*CO_2_ at each treatment was continuously recorded using an IRGA (WMA‐4; PPSystems).

### 2.3 Photosynthesis, respiration, and calcification

Net photosynthesis, respiration, and calcification rates were determined through short‐time incubations of preweighted whole thalli samples (ca. 20 g FW and 30–60 min for photosynthesis and calcification in the light, ca. 40 g FW and 75–120 min for respiration and calcification in the dark). Gently, the excess of water was removed without drying the algae, and the thalli fresh weight (FW) was determined with an electronic balance (Sartorius, 0.1 mg). The weighted algae were then placed in 250‐ml Erlenmeyer flasks filled to the top and sealed to avoid gas diffusion. The Erlenmeyer flasks were placed on an agitation platform to promote water mixture inside the flasks and avoid the trapping of gas bubbles in the irregular forms of the thalli. To control the temperature during the incubations, the flasks were partially immersed in a water bath at the desired temperature, using a thermostatic recirculation system (Julabo HC, Julabo Labortechnik). Two fluorescent tubes were placed above the whole setup, supplying an approximate PAR irradiance of ~50 μmol photons m^−2^ s^−1^. Water samples for dissolved oxygen and total alkalinity (TA) were collected at the beginning and end of the incubations, and the temperature, pH, and salinity measured.

The modified Winkler method was used to determine the dissolved oxygen concentration by direct spectrophotometry (DU 650; Beckman Coulter) following the protocol described in Labasque, Chaumery, Aminot, and Kergoat (2004). Net photosynthesis (NP) and dark respiration (*R*_d_) (µmol O_2_ g^−1^ FW hr^−1^) were calculated from the difference between initial and final concentrations of oxygen, normalized by the incubation time, the volume of the water in each flask, and the fresh weight of the incubated thalli according to the formula;

$$\text{NP}\;\text{or}\;R_{d}\left(\mu \text{mol}\;O_{2}\;g^{-1}\;\text{FW}\;{\text{hr}}^{-1}\right)=\frac{\left(\left[O_{2}\right]\text{fin}-\left[O_{2}\right]\text{in}\right)\times V}{\text{FW}\times T}$$

where [O_2_] is the oxygen concentration (µmol/L), *V* is the volume of the chamber (L), FW is the fresh weight of the incubated algae (g), and *T* is the incubation time in hours.

Gross photosynthesis (GP) was calculated as the sum of the net photosynthesis (NP) and dark respiration (*R*_d_);

$$\text{GP}=\text{NP}+R_{d}$$

Total alkalinity (TA) was determined using the Gran titration method, as in Lewis and Wallace (1998). Subsamples of 80 ml were titrated using an open‐cell automatic titration system, comprised by an Orion 8103SC pH electrode calibrated in the National Bureau of Standards (NBS) scale and an automatic titrator (Metrohm 794 Dosimat), using HCl 0.5 M as the acid titrant. The obtained alkalinity values were corrected using Certified Reference Materials (CRMs, Batch No. 129) supplied by A. Dickson (Scripps Institution of Oceanography). The carbonate chemistry parameters were determined from the measured pH, TA, temperature, and salinity using the software CO_2_SYS (Lewis & Wallace, 1998) with the constants of Mehrbach, Culberson, Hawley, and Pytkowicz (1973) (refitted by Dickson & Millero, 1987). The total alkalinity anomaly technique (Smith & Key, 1975) was used to determine the calcification rates. Light and dark calcification rates (µmol CaCO_3_ g^−1^ FW hr^−1^) were calculated as:

$$G=-\left(\Delta \text{TA}\;v\right)\times {\left(2\Delta t\;\text{FW}\right)}^{-1}$$

where calcification (*G*) is equal to the difference between initial and final TA multiplied by the incubation volume (*v*) and divided by two, the incubation time (Δ*t*) and the fresh weight of the sample (FW).

### 2.4 Statistical analyses

The software package SigmaPlot version 11.0 was used to perform all statistical analysis. Differences in photosynthesis, respiration, and net calcification with temperature were assessed using a one‐way ANOVA. The combined effect of CO_2_ and temperature was tested with a two‐way ANOVA. Normal distribution (Shapiro‐Wilk) and equal variance (Levene's test) were verified. When *p* was significant (*p* < .05), ANOVA was followed by a *post hoc* test for multiple comparisons (Holm‐Sidak). The Pearson correlation coefficient was used to assess the linear dependence between photosynthesis and net calcification.

## 3 RESULTS

**Figure 1:**
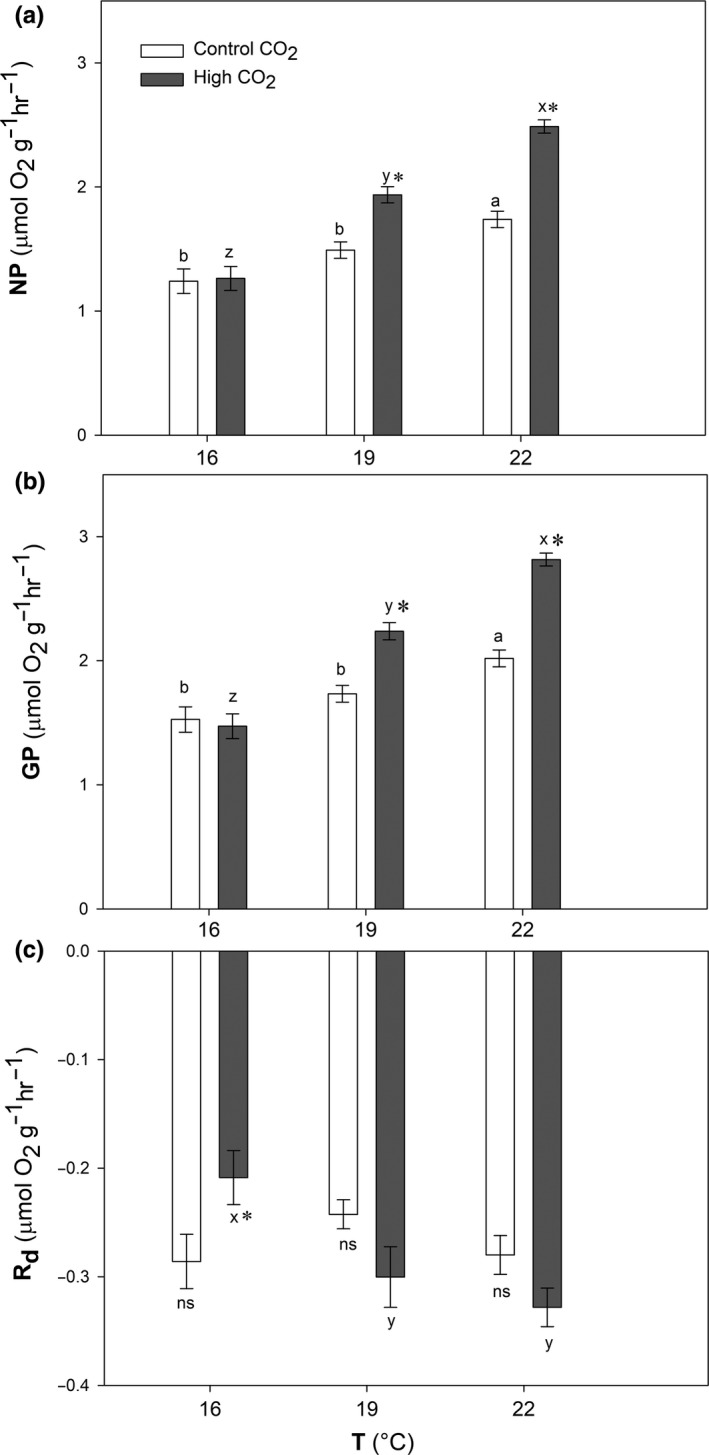
Net photosynthesis (NP) (a), gross photosynthesis (GP) (b), and dark respiration (*R*_d_) (c) (µmol O_2_ g^−1^FW hr^−1^) after 1 month of acclimation to low (16°C), mid (19°C), and high (22°C) temperatures (*T*) and 15 days under control (400 µatm) and high CO_2_ (1,000 µatm) conditions. Mean ± *SE* (*n* = 5), different letters indicate significant differences between temperature levels, and asterisks (*) indicate significant differences between CO_2_ levels and “ns” indicates no significant differences

The positive effect of CO_2_ on the net and gross photosynthesis of *Phymatolithon lusitanicum* was intensified with increasing temperature (Figure 1a,b) (*p* ≤ .001). Increases in photosynthesis with CO_2_ were observed at all temperatures, except at 16°C, where net photosynthesis did not vary between control and high CO_2_ concentration (*p* = .836; Table 1). In contrast, at 22°C we found the highest net production values and the greatest difference between control and high CO_2_ treatments (1.74–2.48 µmol O_2_ g^−1^ FW hr^−1^).

**Table 1:** Summary of the results from the tests performed to assess the effects of temperature (Temp.) and *p*CO_2_ on the respiration, photosynthesis, and calcification of *Phymatolithon lusitanicum*

Source of variation	*df*	Respiration (µmol O_2_ g^−1 ^FW hr^−1^)			Gross photosynthesis (µmol O_2_ g^−1 ^FW hr^−1^)			Net photosynthesis (µmol O_2_ g^−1^FW hr^−1^)			Light calcification (µmol CaCO_3_ g^−1 ^FW hr^−1^)			Dark calcification (µmol CaCO_3_ g^−1 ^FW hr^−1^)		
		MS	*F*	*p*	MS	*F*	*p*	MS	*F*	*p*	MS	*F*	*p*	MS	*F*	*p*
Temp.	2	<0.001	3.692	**.041**	0.0023	49.37	**<.001**	0.0018	59.33	**<.001**	3.748	126.8	**<.001**	0.992	153.7	**<.001**
*p*CO_2_	1	<0.001	0.285	.589	0.0012	25.25	**<.001**	0.0012	40.06	**<.001**	0.212	7.17	**.014**	1.355	209.9	**<.001**
Temp. × *p*CO_2_	2	<0.001	6.314	**.007**	<0.001	12.04	**<.001**	<0.001	10.65	**<.001**	0.198	6.712	**.006**	0.48	74.39	**<.001**

Note

Analysis of variance (ANOVA) *F* tests significant at *p* < .05 are indicated in bold.

Dark respiration increased with temperature, but only under high CO_2_ condition, the highest rates being found at 19 and 22°C (respectively −0.30 and −0.33 µmol O_2_ g^−1^ FW hr^−1^; *p* = .007; Figure 1c). Under control CO_2_, dark respiration was unaffected by temperature and only at the 16°C treatment the respiration rates were higher under control (−0.29 µmol O_2_ g^−1^ FW hr^−1^) than under high CO_2_ conditions (−0.21 µmol O_2_ g^−1^ FW hr^−1^; *p* = .016; Table 1).

**Figure 2:**
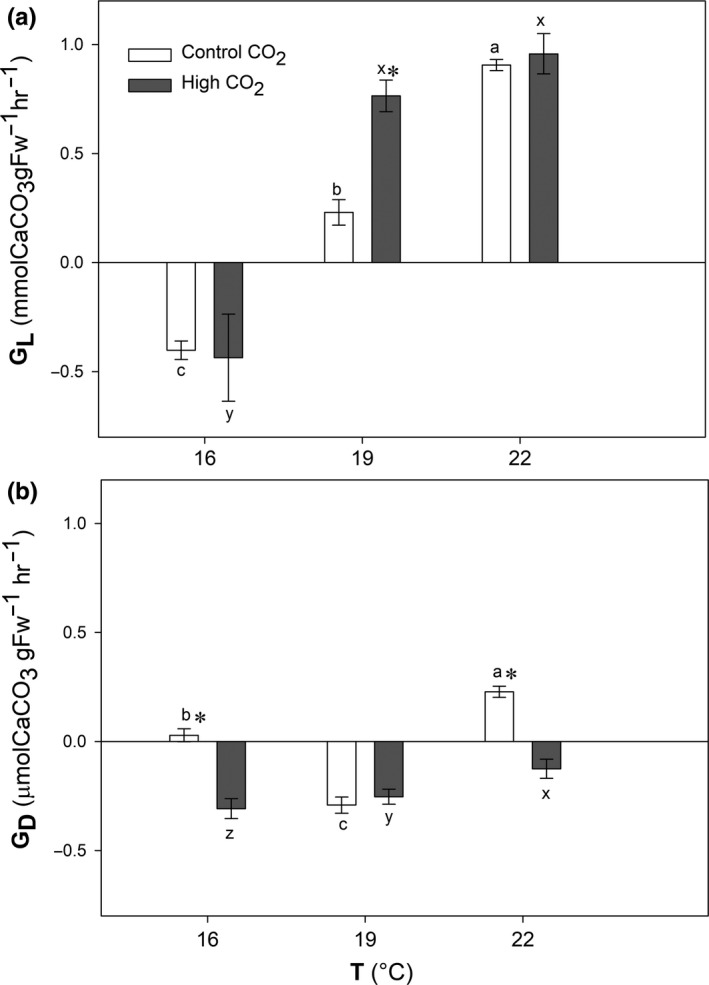
Light (*G*_L_) (a) and dark (*G*_D_) (b) calcification rates (µmol CaCO_3_ g^−1^ FW hr^−1^) of *Phymatolithon lusitanicum* after 1 month of acclimation to low (16°C), mid (19°C), and high (22°C) temperatures (*T*) and 15 days under control (400 µatm) and high CO_2_ (1,000 µatm) conditions. Mean ± *SE* (*n* = 5), different letters indicate significant differences between temperature levels, and asterisks (*) indicate significant differences between CO_2_

Both temperature (*p* < .001) and CO_2_ (*p* = .014) had a significantly positive effect on light calcification (Figure 2a). Under light conditions, algae dissolved at 16°C but under 19 and 22°C algae increased their calcification rates with temperature and CO_2_ (*p* = .006). However, the positive effect of CO_2_ with temperature was only observable at 19°C where light calcification of algae under high CO_2_ conditions (0.76 µmol CaCO_3_ g^−1^ FW^ ^hr^−1^) was threefold higher than under control conditions (0.23 µmol CaCO_3_ g^−1^ FW^ ^hr^−1^; Table 1).

The calcification in the dark (*G*_D_) of *Phymatolithon lusitanicum* increased with temperature only under high CO_2_ conditions (*p* < .001). Under control conditions there was no clear pattern of the effect of temperature on dark calcification, algae dissolved only at 19°C (−0.29 µmol CaCO_3_ g^−1^ FW hr^−1^), and at 22°C dark calcification rates (0.22 µmol CaCO_3_ g^−1^ FW hr^−1^) were sevenfold higher than at 16°C (0.03 µmol CaCO_3_ g^−1^ FW hr^−1^). On the other hand, under high CO_2_, dissolution exceeded calcification in all treatments and dissolution rates decreased with temperature (Figure 2b; Table 1). Algae at 16°C dissolved the most (−0.31 µmol CaCO_3_ g^−1 ^FW hr^−1^) and presented dissolution rates twofold higher than at 20°C (−0.12 µmol CaCO_3_ g^−1^ FW hr^−1^).

**Figure 3:**
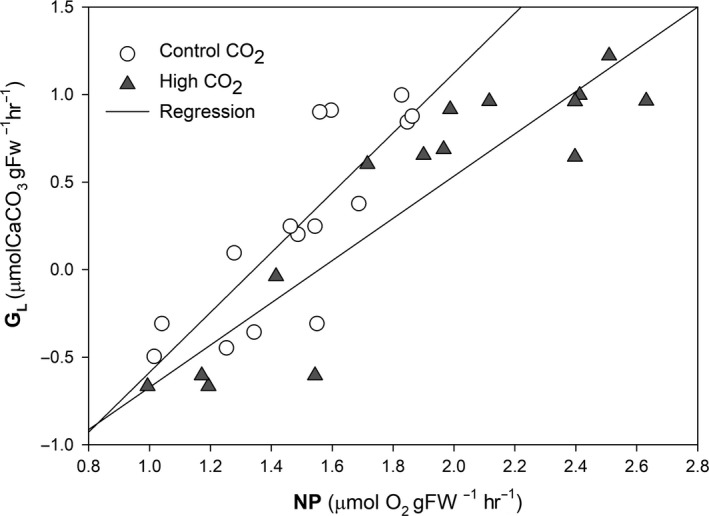
Correlation of light calcification (*G*_L_; µmol CaCO_3_ g^−1^ FW hr^−1^) and net photosynthesis (NP; µmol O_2_ g^−1 ^FW hr^−1^) of *Phymatolithon lusitanicum* after 1 month of acclimation to low (16°C), mid (19°C) and high (22°C) temperatures and 15 days of high CO_2_ (1,000 µatm). Mean ± *SE* (*n* = 5)

Net calcification was positively correlated with net photosynthesis in a very significant manner, both under control (Pearson correlation; *R* = 0.827; *p* < .001) and high CO_2_ conditions (*R* = 0.910; *p* < .001; Figure 3). Under high CO_2_ conditions, and while maintaining a strong positive correlation, calcification rates were lower than at control conditions for identical photosynthetic rates.

The mean seawater temperature and parameters of the carbonate system in each treatment under light and dark conditions are depicted in Table 2.

**Table 2:** Carbonate system parameters in each treatment

Treatment	Salinity	*T* (°C)	pH	TA (µmol/kg)	DIC (µmol/kg)	*Ω*_AR_
Dark incubations						
LOW/control CO_2_	33.05 ± 0.14	15.87 ± 0.02	8.09 ± 0.01	2,407.16 ± 8.86	2,220.87 ± 9.06	2.21 ± 0.03
LOW/high CO_2_	33.16 ± 0.08	15.90 ± 0.00	7.55 ± 0.00	2,526.75 ± 4.99	2,534.57 ± 3.54	0.75 ± 0.00
MID/control CO_2_	33.60 ± 0.23	17.6 ± 0.06	8.09 ± 0.01	2,378.17 ± 13.14	2,181.22 ± 15.73	2.31 ± 0.09
MID/high CO_2_	33.48 ± 0.17	17.64 ± 0.05	7.89 ± 0.01	2,409.99 ± 9.56	2,295.69 ± 14.29	1.57 ± 0.04
HIGH/control CO_2_	33.30 ± 0.15	22.00 ± 0.00	8.04 ± 0.00	2,311.13 ± 9.58	2,115.48 ± 10.06	2.34 ± 0.01
HIGH/high CO_2_	33.44 ± 0.09	22.00 ± 0.00	7.88 ± 0.02	2,449.04 ± 27.80	2,317.82 ± 31.85	1.79 ± 0.06
Light incubations						
LOW/control CO_2_	33.75 ± 0.03	15.40 ± 0.04	8.37 ± 0.02	2,454.69 ± 5.02	2,111.06 ± 13.10	3.79 ± 0.14
LOW/high CO_2_	33.85 ± 0.05	15.65 ± 0.05	8.20 ± 0.00	2,428.59 ± 26.59	2,183.49 ± 22.58	2.78 ± 0.05
MID/control CO_2_	33.55 ± 0.17	17.60 ± 0.04	8.43 ± 0.00	2,365.59 ± 31.62	1,971.85 ± 28.17	4.30 ± 0.06
MID/high CO_2_	34.00 ± 0.10	18.45 ± 0.05	8.03 ± 0.00	2,294.99 ± 2.18	2,120.79 ± 3.08	2.06 ± 0.01
HIGH/control CO_2_	33.46 ± 0.08	22.00 ± 0.00	8.25 ± 0.03	2,329.37 ± 2.50	2,016.83 ± 19.26	3.53 ± 0.18
HIGH/high CO_2_	33.50 ± 0.04	22.00 ± 0.00	7.81 ± 0.07	2,322.32 ± 7.49	2,216.17 ± 28.67	1.54 ± 0.23

Note

Salinity, temperature (*T*), total alkalinity (TA), and pH (NBS scale) were measured, while dissolved inorganic carbon (DIC) and saturation state of seawater with respect to aragonite (*Ω*_AR_) were calculated. The values are expressed as means ± *SE* (*n* = 5).

## 4 DISCUSSION

We found a positive interaction between CO_2_ and temperature for all the metabolic rates of *Phymatolithon lusitanicum*, with temperature being the factor that contributed the most to the differences observed on photosynthesis, light calcification, and respiration of the algae, while CO_2_ was the determinant factor on dark calcification. The positive coupling between photosynthesis and calcification is thus expected to be accentuated under high CO_2_ conditions. However, in this scenario, photosynthesis will be favored over calcification, with potential effects on the blue carbon fixing capacity of the rhodolith beds from southern Portugal.

### 4.1 Effect of temperature and high CO_2_ on photosynthesis

Both net and gross photosynthesis of *Phymatolithon lusitanicum* increased with temperature and this increase was amplified under high CO_2_ conditions. At the mid and high temperature treatments, photosynthetic rates increased under high CO_2_ conditions and only at 16°C there was no *p*CO_2_ effect. Our results show that in a high CO_2_ scenario with a drastic increase of temperature, such as a heat wave, the photosynthetic rates of these algae will be positively affected by the combination of these two factors.

In a 10‐day experiment, Vásquez‐Elizondo and Enríquez (2016) found that the photosynthetic rates of the three tropical coralline algae species *Neogoniolithon* sp. (rhodolith), *Lithothamnion* sp. (crustose coralline algae) and *Amphiroa tribulus*, in Puerto Morelos, Mexico, were unaffected by high CO_2_ and concluded that elevated temperature is a stronger threat to coralline algae than ocean acidification (OA). In contrast, in a 1‐month experiment, Noisette, Egilsdottir, et al. (2013) confirmed that the gross production of the subtidal and temperate rhodolith species *Lithothamnion corallioides* slightly increased with CO_2_ (1,000 μatm), while in a 3‐month experiment Noisette, Duong, et al. (2013) also found that the gross photosynthesis of the rhodolith *Lithothamnion corallioides* increased with CO_2_ (550 μatm) but it was unaffected by temperature. However, the high temperature used in their experiment (19°C) was a lower temperature than the temperature used in this study (22°C). As in Noisette, Duong, et al. (2013), no differences were found in the present study between the 16 and 19°C treatments under control conditions, and the effect of temperature was only evident at 22°C.

Vásquez‐Elizondo and Enríquez (2016) also suggested that the undetected effect of temperature on the photosynthesis of algae may be related to the low light levels used during experiments to properly mimic natural conditions. This agrees with the results obtained by Martin et al. (2013) in a long‐term experiment (1 year) with the Mediterranean crustose *Lithophyllum cabiochae*, where it was found that under ambient irradiance the photosynthesis was unaffected by temperature in most seasons but decreased under elevated CO_2_ in summer, autumn, and winter. The authors concluded that the effect of high CO_2_ on the photosynthesis of coralline algae depends on the irradiance level used in the experiments and that it is critical to consider the interaction of seasonal changes of temperature and also irradiance during OA experiments.

In a previous long‐term experiment with *Phymatolithon lusitanicum* (Sordo et al., 2018), the authors also observed no effect of high CO_2_ under low irradiance (8 μmol photons m^−2^ s^−1^). However, when irradiance was increased during the short‐time incubations (to a maximum of 200 μmol photons m^−2^ s^−1^), the differences among treatments increased proportionally to CO_2_ concentration.

### 4.2 Effect of temperature and high CO_2_ on respiration

Under high CO_2_ conditions, respiration increased at 19 and 22°C, while under control conditions it was unaffected by temperature. In previous studies with temperate coralline algal species, Sordo et al. (2016) and Noisette, Duong, et al. (2013) found that the respiration rates of the rhodolith species *Phymatolithon lusitanicum* and *Lithothamnium corallioides*, were unaffected by high CO_2_ but increased with temperature, respectively. On the other hand, Martin et al. (2013) found that the respiration rates of the crustose *Lithophyllum cabiochae* were unaffected by elevated CO_2_ but showed a trend to increase with temperature, with the exception of summer where *R*_d_ decreased with increasing temperature and CO_2_. In a previous study with the tropical rhodolith *Neogoniolithon* sp., the crustose *Lithothamnion* sp. and the articulated *Amphiroa tribulus*, Vásquez‐Elizondo and Enríquez (2016) found that respiration increased with temperature, but after 10 days under high CO_2_ and temperature conditions, algae decreased their respiration rates.

### 4.3 Effect of temperature and high CO_2_ on calcification

After 1 month of treatment, *Phymatolithon lusitanicum* calcification increased with temperature. Even if there was a synergistic interaction between temperature and CO_2_, the effect of high CO_2_ on the net calcification of algae was significant only at 19°C. The increase of net calcification with temperature can be explained by the Boltzmann–Arrhenius function, which shows that there is a temperature dependence of the underlying metabolic rates (growth rate, photosynthesis, and respiration) and carbon allocation efficiency of primary producers with exponential growth until a physiological tipping point is reached (García‐Carreras et al., 2018). Our results show, in addition, that this increase was accentuated under high CO_2_ conditions. Previous research found that under high CO_2_, algae survived by increasing their calcification rates to compensate the dissolution that occurs during the night (Kamenos et al., 2013). Even if acidified algae calcify more to compensate the drop of pH, they are still under a constant dissolution/calcification process and in the long term are expected to grow less (Dupont & Pörtner, 2013; see Sordo et al., 2018). Kamenos et al. (2013) also found that when this pH change occurs abruptly there is an additional weakening of the calcite skeleton.

In a previous 21‐day study with the tropical crustose coralline algae *Hydrolithon onkodes*, Johnson and Carpenter (2012) also found that temperature and CO_2_ had a significant synergistic effect on the algae's net calcification. Even if *H. onkodes* responded under moderately elevated CO_2_ by increasing its calcification rates, this response was variable and unaligned with the highest calcification at ambient temperature (26°C). The authors also found that under high CO_2_ and temperature the protective function of the calcified thallus was reduced, becoming more susceptible to grazing with important cascading community effects on the stability of coral reefs and their associated biodiversity (Johnson & Carpenter, 2012).

On the other hand, Büdenbender, Riebesell, and Form (2011) found, in two separate experiments (1‐month long) (at 6.8°C and 0 μmol photons m^−2^ s^−1^ for winter vs. 9°C and 6.8 μmol photons m^−2^ s^−1^ for summer), that the crustose arctic species *Lithothamnion glaciale* decreases its calcification rates with high CO_2_ (815, 975, 1,570 ppm), and only in summer the algae were able to increase (815 ppm) or maintain (975 ppm) their net calcification rates.

In this study, after 1 month, *Phymatolithon lusitanicum* calcification in the dark under control conditions showed positive values at 16 and 22°C but negative values under 19°C. Many nongeniculate temperate rhodoliths are low light adapted, and there is evidence that these algae can grow for long periods in the dark (Wilson, Blake, Berges, & Maggs, 2004). Dark calcification is likely sourced by an accumulation of energy during periods of light, and there is evidence that some coralline algae can calcify in the dark (reviewed in McCoy & Kamenos, 2015). However, this mechanism could also explain the growth decrease and dissolution under stressful conditions (McCoy & Kamenos, 2015). This agrees with the results from this study, where dissolution surpassed calcification in the dark under stressful conditions. Under an intermediate temperature and control CO_2_, algae were stressed and presented low light calcification rates while under dark conditions dissolution surpassed calcification. According to Hurd et al. (2011), even under control CO_2_ conditions, dissolution can occur in the dark due to the reduced pH in the diffuse boundary layer (DBL) between the algal surface and surrounding water.

The negative light calcification rates observed at 16°C suggest that this light level was probably too high for the low temperature tested. Under natural conditions at low temperatures, such as 16°C, these algae are normally exposed to lower irradiances than 50 μmol photons m^−2^ s^−1^. The results observed could be related to a physiological stress response by the algae.

The potentially stressful conditions were also reflected on the loss of pigmentation of the algae at the end of the experiment (personal observations). This was probably related to the high light levels used during the experiment. Even if values close to 50 μmol photons m^−2^ s^−1^ were occasionally recorded on the field, most of the time these algae are exposed to low light and temperature conditions. In a previous long‐term experiment with *P. lusitanicum*, Sordo et al. (2018) observed a loss of pigmentation, especially accentuated at the acidified algae, but only after 20 months of high CO_2_ conditions. During these experiments, algae were cultivated at low light and temperature conditions, and these conditions increased their resilience to a long‐term exposure to high CO_2_.

In the present short‐term study, under high CO_2_ conditions dissolution surpassed calcification in all treatments and decreased with temperature. This agrees with previous studies (Kamenos et al., 2013; Martin et al., 2013; Martin & Gattuso, 2009; Noisette, Duong, et al., 2013; Noisette, Egilsdottir, et al., 2013) where dark calcification of coralline algae exposed at high CO_2_ was positively affected by temperature. Kamenos et al. (2013) observed that under dark conditions the rhodolith *Lithothamnion glaciale* was able to calcify under control conditions but dissolved under high CO_2_ conditions (pH 7.70; ~1,081 μatm). Also, Noisette, Egilsdottir, et al. (2013) in a 1‐month experiment found that the calcification rates of the temperate crustose coralline algae *Lithophyllum incrustans* (both under light and dark conditions) and the rhodolith *Lithothamnion corallioides* (only under light conditions) decreased under elevated *p*CO_2_ (750–1,000 μatm). In other studies, under different temperatures and CO_2_ concentrations, interactions between both factors have been found like in Noisette, Duong, et al. (2013) where in a 3‐month experiment the dark calcification of the temperate rhodolith *Lithothamnion corallioides* increased with temperature (from 10 to 16°C) but decreased with CO_2_ (1,000 μatm). Also, Martin Cohu Vignot Zimmerman and Gattuso (2013) found in a 1‐year experiment that the dark calcification of the Mediterranean crustose algae *Lithophyllum cabiochae* was negatively affected by increasing temperature, but only under high CO_2_ conditions. Likewise, Martin and Gattuso (2009) found a higher sensitivity of *Lithophyllum cabiochae* to warming under elevated CO_2_. In addition, both respiration and calcification release CO_2_, thus decreasing the pH of seawater. The inhibition of calcification in the dark can be further amplified by the release of respiratory CO_2_ (Hurd et al., 2009; McCoy & Kamenos, 2015).

### 4.4 Photosynthesis and calcification

Even if it is widely accepted that calcification and photosynthesis are interconnected (Hurd et al., 2009), the details of this coupling under high CO_2_ conditions are not yet fully understood (Johnson & Carpenter, 2012). Vásquez‐Elizondo and Enríquez (2016) proposed a physiological model to estimate the carbonate production of coralline algae under thermal stress. The authors found a linear correlation between calcification and respiration but a nonlinear coupling between algal calcification and photosynthesis. In addition, they demonstrated that coralline algae are more sensitive to photodamage under increasing temperature. So, differences in results between species can also be explained by the natural habitat where they live and their specific photobiology. However, this model does not take into account the lowering of pH on calcification, and it is adapted to highly illuminated habitats in the tropics, such as coral reefs, which are strongly dependant on irradiance changes (Vásquez‐Elizondo & Enríquez, 2016).

In this study, we observed that the relationship between photosynthesis and calcification was positive at the two CO_2_ levels, but calcification rates under high CO_2_ were lower than under control conditions for identical photosynthetic rates, even if the rates of both processes generally increased with temperature and CO_2_. Acidified algae increase their calcification rates to compensate the dissolution as a consequence of the lowering of the saturation state of water. We hypothesize that in a short term, algae under high temperature and CO_2_ conditions calcify more but also dissolve more and therefore in the long term will grow less than control algae. A previous OA long‐term experiment with *Phymatolithon lusitanicum* (Sordo et al., 2018) supports these observations. After 11 months, algae increased both the photosynthetic and calcification rates under high CO_2_. However, after 20 months, there was a decrease of both rates. Calcification was specially compromised so that at the end of the experiment acidified algae presented the lowest accumulated growth.

## 5 CONCLUSIONS

The response of *Phymatolithon lusitanicum* to increasing temperature is amplified by increasing CO_2_. Temperature has a positive effect on the photosynthesis, respiration, and calcification of *Phymatolithon lusitanicum,* and this effect is intensified under high CO_2_ conditions. The results from this study suggest that it is important to consider the interactive effects of temperature and high CO_2_ on the main physiological processes as already suggested by Johnson and Carpenter (2012). This interaction could partially explain the divergences observed among OA experimental studies with coralline algae. The integration of additional abiotic stressors, such as light and temperature in OA experiments with coralline algae will increase our understanding of the basic relationship of photosynthesis and calcification as a function of environmental parameters (McCoy & Kamenos, 2015).

In a short term and under increasing temperature and *p*CO_2_, *Phymatolithon lusitanicum* may increase its photosynthetic, calcification, and respiration rates. However, algae may not be able to sustain high rates in the long term. The increase of the respiration rates is expected to increase the dissolution of algae in the dark and probably their susceptibility to grazing. The coupling between calcification and photosynthesis will be intensified. However, under high CO_2_, this coupling will favor photosynthesis over calcification, and in the long term this could have a significant effect on the blue carbon fixing capacity of the rhodolith beds from southern Portugal. Higher energetic costs are expected to have a negative effect on the growth of the algae and the long‐term exposure to high temperature and CO_2_ may decrease the resilience of *Phymatolithon lusitanicum*.

Under the current PAR irradiances and temperatures observed in the field in southern Portugal, the metabolic rates of *Phymatolithon lusitanicum* could be unaffected by increasing high CO_2_ in the near future. However, under rapid increases of temperature, such as the heat wave recorded in summer of 2013, *P. lusitanicum* will increase their metabolic rates, and the effect of high CO_2_ will be accentuated with temperature. In addition, we expect the results to change with light irradiance. Because of this, further research where these three physical variables (temperature, *p*CO_2_, and irradiance) are considered and where photosynthesis, calcification, and respiration are addressed simultaneously is necessary to elucidate the future resilience of maërl beds to sporadic heat waves under increasing *p*CO_2_.

## CONFLICT OF INTEREST

None declared.

## AUTHOR CONTRIBUTIONS

LS conceived, designed, and performed the experiments, analyzed and interpreted the data, wrote the paper, prepared the figures and tables, reviewed the paper, and approved the final version. RS contributed with the interpretation of data and revision of the manuscript. IB contributed with the interpretation and representation of data and the revision of the manuscript. JS contributed with the design of the work, the interpretation of data, and the revision of the initial drafts of the manuscript giving a final approval to the version submitted.

## Data Availability

Data available at https://doi.org/10.5061/dryad.6d5d302.
